# Errata

**Published:** 2013-07-12

## Vol. 62, No. 23

In the report, “Nationwide Rubella Epidemic — Japan, 2013” an error occurred in the first sentence of the first full paragraph on page 460. That sentence should read, “In the current outbreak, males aged 20–39 years, many of whom had not been vaccinated in the initial rubella vaccination program for male junior high students offered only in clinics and hospitals, have accounted for 46% of reported cases.”

## Vol. 62, No. 25

In the report, “HIV and Syphilis Infection Among Men Who Have Sex with Men — Bangkok, Thailand, 2005–2011,” an error occurred in the table of on page 519. In the median (range) row, the number under clients should be **27**.

